# The Association Between Possible Sarcopenia and Delirium Onset in Older Patients With Acute Stroke

**DOI:** 10.7759/cureus.86993

**Published:** 2025-06-29

**Authors:** Kaori Shiozaki, Ayano Nagano, Mariko Hanaoka, Yuki Uchiyama, Kazuhisa Domen, Tetsuo Koyama

**Affiliations:** 1 Department of Nursing, Nishinomiya Kyoritsu Neurosurgical Hospital, Nishinomiya, JPN; 2 Department of Nutrition, Nishinomiya Kyoritsu Neurosurgical Hospital, Nishinomiya, JPN; 3 Department of Rehabilitation Medicine, School of Medicine, Hyogo Medical University, Nishinomiya, JPN; 4 Department of Rehabilitation Medicine, Nishinomiya Kyoritsu Neurosurgical Hospital, Nishinomiya, JPN

**Keywords:** geriatric medicine, nursing, nutrition, retrospective research, risk assessment

## Abstract

Introduction

Delirium is a common and serious complication among older adults hospitalized with acute stroke, and it is associated with increased morbidity, mortality, and prolonged hospital stays. Sarcopenia, characterized by a decline in muscle mass and strength, has emerged as a potential risk factor for delirium. However, there has been scarce research exploring this relationship in stroke populations. This study aimed to investigate the association between possible sarcopenia and the onset of delirium in older patients with acute stroke.

Methods

We conducted a retrospective cohort study of patients aged 65 years and older admitted to the Stroke Care Unit of a single institution between April 2020 and March 2021. Possible sarcopenia was defined using the AWGS2019 criteria based on grip strength and calf circumference. Delirium was diagnosed according to the Diagnostic and Statistical Manual of Mental Disorders, Fifth Edition (DSM-5) criteria by a certified dementia care team. Clinical characteristics and known delirium risk factors were extracted from medical records. Logistic regression analysis was performed to identify independent predictors of delirium.

Results

Of the 543 patients screened, 486 were included in the final analysis. Possible sarcopenia was identified in 187 patients (38.4%). Delirium occurred in 46 patients (9.4%), and its incidence was significantly higher in the sarcopenia group (15.0%) than in the non-sarcopenia group (6.0%, p=0.001). Multivariate logistic regression analysis revealed that possible sarcopenia was independently associated with delirium onset [odds ratio (OR): 1.98, 95% confidence interval (CI): 1.01-3.91, p=0.048], second only to a history of delirium.

Conclusions

Our findings suggest that possible sarcopenia is a significant and independent predictor of delirium in older patients with acute stroke. Early identification of sarcopenia may facilitate targeted interventions to reduce delirium risk and improve clinical outcomes in this vulnerable population.

## Introduction

Delirium is a pathological condition characterized by acute and transient impairments in attention, cognition, and level of consciousness, affecting up to 50% of hospitalized older adults with acute stroke [1-5]. It has been associated with increased mortality, prolonged hospital stays, and a significantly higher risk of institutionalization [6]. It is also linked to an increased rate of cognitive decline among older patients with dementia [7]. In stroke patients, delirium contributes to extended hospital stays, increased mortality, and poor functional outcomes [3,8]. Given its clinical impact, recent stroke guidelines, in both Japan and internationally, emphasize the importance of routine delirium screening and early intervention. The Japan Stroke Society Guideline 2021 for the Treatment of Stroke [9] recommends regular assessment of mental symptoms, including delirium. Similarly, the National Clinical Guideline for Stroke for the UK and Ireland [10] stresses the need for systematic delirium screening and multidisciplinary vigilance throughout hospitalization.

Sarcopenia, an age-related skeletal muscle disease characterized by reduced muscle mass, strength, and physical function [11], is associated with an increased risk of falls, fractures, physical disability, and hospitalization [12]. However, sarcopenia is not exclusively age-related; it may also result from multiple factors, including poor nutrition, physical inactivity, comorbidities, and iatrogenic causes [11]. Among older adults hospitalized with acute stroke, many already exhibit sarcopenia at the time of admission [13,14]. This pre-existing sarcopenia has a significant impact on both short-term outcomes and long-term recovery and prognosis [14]. Early identification of sarcopenia is considered essential for optimizing rehabilitation and minimizing adverse outcomes in this population. Accordingly, sarcopenia assessment at the time of hospital admission should be regarded as a critical component of comprehensive stroke care in older adults.

Both sarcopenia and delirium are prevalent and impactful complications following stroke. Previous studies have shown that sarcopenia and delirium are independently associated in older adults admitted to acute care settings. For instance, Bellelli et al. reported that sarcopenia, as defined by the European Working Group on Sarcopenia in Older People criteria, was independently associated with the development of delirium in hospitalized geriatric patients [15]. In addition, calf circumference (CC), a surrogate marker for muscle mass, has also been linked to delirium risk [16]. Despite these findings, few studies have specifically examined this relationship in stroke populations or explored its implications for clinical outcomes. Clarifying this relationship in the context of stroke is of particular importance, as both conditions are known to adversely affect recovery. Therefore, this study aims to determine the independent association between possible sarcopenia and delirium onset in older patients hospitalized with acute stroke. Understanding this association may help devise targeted prevention strategies and enhance the management of vulnerable patients in the acute stroke setting. We hypothesize that sarcopenia increases the risk of delirium in this population.

## Materials and methods

Patients

This study targeted acute stroke patients aged 65 years and above who were admitted to the Stroke Care Unit (SCU) at Nishinomiya Kyoritsu Neurosurgical Hospital from April 2021 to March 2022. Patients whose grip strength or CC could not be measured within the first three days of admission were excluded. Data were retrospectively collected from medical records. Baseline characteristics obtained included age, sex, height, weight, BMI, and risk factors for delirium onset. Delirium risk factors were assessed based on the checklist defined by the Ministry of Health, Labour and Welfare of Japan for the “Delirium High-Risk Care Fee” [17]. Specifically, the following risk factors were extracted from medical records: history of delirium, dementia, heavy alcohol use (defined as an average daily intake of pure alcohol exceeding 60 g), use of benzodiazepine medications at the time of admission, and scheduled surgeries. The duration of hospital stay was also recorded.

Ethical approval

This study was approved by the Ethics Committee of Hyogo Medical University (approval no. 4506) and conducted in accordance with the Declaration of Helsinki. Informed consent was obtained using an opt-out method.

Diagnosis of sarcopenia

At the SCU, grip strength and CC measurements are part of the nutritional assessment and are used to screen sarcopenia upon admission. Grip strength was measured within three days of hospitalization using a Smedley hand dynamometer (Matsuyoshi Medical Equipment Grip Strength Meter DX model, Matsuyoshi Medical Instruments Co., Ltd., Tokyo, Japan). Two alternating measurements were taken for each hand, with the highest value recorded as the final grip strength. CC was measured at the point of maximum circumference of the calf with the knee joint at a 90-degree angle using a tape measure, and the larger value between the two legs was used. According to the Asian Working Group for Sarcopenia 2019 (AWGS2019) criteria [18], sarcopenia was considered possible if the values were below 34 cm in circumference for males and 33 cm for females, and below 28 kg of grip strength for males and 18 kg for females. The AWGS2019 criteria for diagnosing sarcopenia include an assessment of physical function; however, due to difficulties in assessing physical function in acute stroke patients, who may suffer from motor function impairments such as paralysis, this component was not assessed in our study.

Delirium assessment

Delirium was assessed based on the diagnostic criteria of the fifth edition of the Diagnostic and Statistical Manual of Mental Disorders (DSM-5) [19]. Delirium was diagnosed when all of the following DSM-5 criteria (A-E) were fulfilled: (A) disturbance in attention and awareness; B) the disturbance develops over a short period (usually hours to a few days), represents a change from baseline, and tends to fluctuate in severity during the course of a day; C) additional disturbances in cognition (e.g., memory, disorientation, language, visuospatial ability, or perception); D) the disturbances in A and C are not better explained by another pre-existing, established, or evolving neurocognitive disorder and do not occur in the context of severely reduced arousal, such as coma; and E) there is evidence from history, physical examination, or laboratory findings that the disturbance is a direct physiological consequence of another medical condition, substance intoxication or withdrawal, exposure to a toxin, or multiple etiologies. Delirium cases during hospitalization were identified by a dementia care team consisting of certified dementia care nurses and physicians, and confirmed based on entries in the medical records.

Statistical analysis

Continuous variables are presented as mean ± standard deviation (SD), and ordinal variables as median [interquartile range(IQR)]. Categorical variables are expressed as numbers (percentages). For group comparisons, the chi-squared test or Fisher’s exact test was used for categorical variables. For continuous variables, the independent t-test was applied when the data were normally distributed, and the Mann-Whitney U test was used when distributions were non-normal. Participants were categorized into sarcopenia and non-sarcopenia groups, and univariate analyses were first conducted to examine group differences. In this analysis, effect sizes were calculated as Pearson’s r for continuous variables and φ (phi) coefficients for categorical variables, as appropriate. In addition, the association between known risk factors for delirium and its occurrence in the present dataset was assessed using Fisher's exact test. Subsequently, a multiple logistic regression analysis was performed to evaluate the contribution of sarcopenia to the onset of delirium. Given the sample size in our study, it was not feasible to include a large number of variables in the multivariate logistic regression model. Therefore, we prioritized the inclusion of variables that are clinically recognized as strong risk factors for delirium. To ensure both statistical validity and clinical relevance, we selected key items from the Japanese national “Delirium High-Risk Care Fee” checklist. Nonetheless, we acknowledge the possibility of residual confounding due to unmeasured variables. Statistical significance was set at p<0.05.

## Results

**Figure 1:**
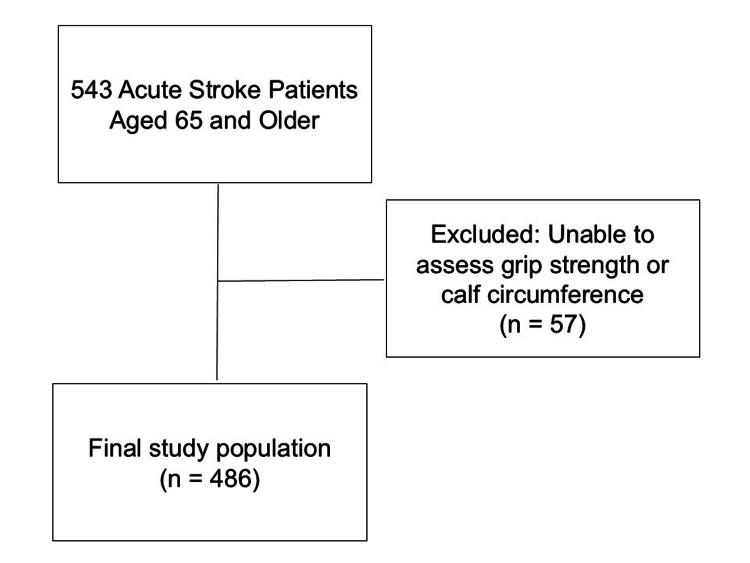
Flow diagram of participant selection A total of 543 acute stroke patients aged 65 years and older were initially screened. Fifty-seven patients were excluded due to the inability to assess grip strength or calf circumference within the first three days of admission. The final study population included 486 patients

Of the 543 acute stroke patients aged 65 years and older admitted to the SCU, 57 were excluded due to difficulties in assessing grip strength or CC, leaving 486 patients for analysis. All other variables, including demographic, clinical, and outcome data, were complete and included in the statistical analysis (Figure 1).

Baseline characteristics of the cohort are presented in Table 1. Possible sarcopenia was identified in 187 participants (38.4%). During hospitalization, 46 patients (9.4%) developed delirium. Delirium occurred significantly more frequently in the sarcopenia group (28 cases; 15.0%) than in the non-sarcopenia group (18 cases; 6.0%) (p=0.001). Significant differences were also observed between groups regarding age (p<0.001), BMI (p<0.001), gender (p<0.001), history of delirium (p=0.022), and presence of dementia (p<0.001), all of which were more common in the sarcopenia group.

**Table 1:** Patient characteristics ^†^χ² test; ^‡^Fisher’s exact test (effect size not reported for Fisher’s exact test and shown as NA) BMI: body mass index; IQR: interquartile range; SD: standard deviation

Variable	Total (n=486)	Sarcopenia (n=187)	Non-sarcopenia (n=299)	P-value	Effect size
Age, years, mean ± SD	79.8 ± 8.9	84.1 ± 8.4	77.1 ± 8.0	<0.001	0.39
Male, n (%)	272 (55.9)	76 (40.6)	196 (65.6)	<0.001^†^	0.24
BMI, kg/m², mean ± SD	22.6 ± 3.5	20.3 ± 2.6	24.0 ± 3.3	<0.001	0.51
Low grip strength, n (%)	322 (66.2)	187 (100)	135 (45.2)	<0.001^†^	0.56
Grip strength, male, kg, median (IQR)	25.1 (20.0-30.2)	18.0 (12.0-23.0)	29.0 (24.0-33.0)	<0.001	0.19
Grip strength, female, kg, median (IQR)	11.9 (7-17)	8.0 (5.0-12.0)	17.0 (11.5-19.0)	<0.001	0.26
Low calf circumference, n (%)	227 (46.7)	187 (100)	40 (13.3)	<0.001^†^	0.72
Calf circumference, male, cm, median (IQR)	34.3 (32.0-36.6)	30.7 (29.0-32.0)	36.0 (34.5-38.0)	<0.001	0.30
Calf circumference, female, cm, median (IQR)	31.5 (29.0-34.0)	29.5 (27.0-31.0)	34.0 (3.0-36.0)	<0.001	0.10
Length of hospital stay, days, median (IQR)	17 (11-27)	21 (14-31)	15 (10-23)	<0.001	0.75
Delirium during hospitalization, n (%)	46 (9.8)	28 (15.0)	18 (6.0)	0.001^‡^	NA
Delirium risk factors, n (%)					
History of delirium	14 (2.8)	10 (5.3)	4 (1.3)	0.022^‡^	NA
Dementia	89 (18.3)	64 (34.2)	25 (8.4)	<0.001^†^	0.32
Benzodiazepine use	70 (14.4)	30 (16.0)	40 (13.4)	0.496^†^	0.03
Scheduled surgery	28 (5.7)	10 (5.3)	18 (6.0)	0.757^†^	0.01
Heavy alcohol use	8 (1.6)	2 (1.1)	6 (2.0)	0.717^‡^	NA

Table 2 shows the association between delirium occurrence and its individual risk factors. Significant associations with delirium were observed for all factors except heavy alcohol use, scheduled surgery, and benzodiazepine use.

**Table 2:** Association between delirium and risk factors Fisher’s exact test was used for all categorical comparisons due to small sample sizes or expected cell counts <5

Variable	Total (n =486), n (%)	Delirium (n=46), n (%)	No delirium (n=440), n (%)	P-value
History of delirium	14 (2.8)	6 (13.0)	8 (1.8)	0.001
Dementia	89 (18.3)	17 (37.0)	72 (16.4)	0.002
Benzodiazepine use	70 (14.4)	11 (23.9)	59 (13.4)	0.074
Scheduled surgery	28 (5.7)	4 (8.7)	24 (5.5)	0.325
Heavy alcohol use	8 (1.6)	1 (2.2)	7 (1.6)	0.551
Age ≥70 years	419 (86.2)	43 (93.5)	376 (85.5)	0.177

Table 3 presents the results of the logistic regression analysis, examining the influence of possible sarcopenia and other covariates on delirium during hospitalization. Possible sarcopenia was identified as a statistically significant predictor [odds ratio (OR): 1.98, 95% confidence interval (CI): 1.010-3.910, p=0.048], ranking second after a history of delirium.

**Table 3:** Multivariate Logistic Regression Analysis for Delirium Occurrence VIF: variance inflation factor, used to assess multicollinearity among explanatory variables in the regression model.

Variable	Odds ratio	95% CI	P-value	VIF
Possible sarcopenia	1.980	1.01-3.910	0.048	1.134
History of delirium	5.180	1.510-17.80	0.008	1.126
Dementia	1.890	0.912-3.920	0.087	1.167
Benzodiazepine use	1.870	0.853-4.100	0.118	1.030
Scheduled surgery	1.270	0.377-4.270	0.701	1.036
Heavy alcohol use	0.737	0.0641-8.480	0.807	1.089
Age ≥ 70 years	1.440	0.416-4.990	0.565	1.062

## Discussion

This study examined the association between the presence of potential sarcopenia at admission and the development of delirium during hospitalization in acute stroke patients aged 65 years and older admitted to the SCU. Two key findings emerged from the analysis. First, patients with potential sarcopenia at admission were more likely to develop delirium during their hospital stay. Second, potential sarcopenia was identified as an independent predictor of delirium onset during the acute phase of hospitalization.

The observed association between potential sarcopenia at admission and an increased risk of developing delirium may be explained by several underlying mechanisms. Sarcopenia often reflects a decline in overall physiological reserve and is frequently accompanied by malnutrition, inflammation, and immobility [11]. In the acute phase of stroke, these vulnerabilities may be further exacerbated by neurological deficits, prolonged bed rest, and acute stress responses, making patients with sarcopenia particularly susceptible to delirium. Inflammation is a key contributor to sarcopenia, and proinflammatory cytokines such as TNF-α, IL-6, and IL-1 are frequently elevated in individuals with sarcopenia [20]. These same inflammatory mediators have also been implicated in the pathogenesis of delirium [21], suggesting that inflammation may serve as a common pathway linking sarcopenia and delirium. Furthermore, recent studies have proposed the existence of a muscle-brain axis [22,23], wherein muscle degradation contributes to neuroinflammation and impaired cognitive function. These findings support the notion that sarcopenia is not merely a physical condition but also a marker of systemic and neurological vulnerability, particularly in older adults with acute stroke.

The identification of potential sarcopenia as an independent predictor of delirium further highlights its clinical significance. In this study, only a history of delirium and the potential for sarcopenia were independently associated with the onset of delirium. This finding implies that the presence of sarcopenia contributes to the onset of delirium beyond other commonly known factors, such as age, history of alcohol abuse, or pre-existing cognitive impairment [24-26]. It suggests that sarcopenia may play a more direct role in the pathophysiology of delirium or serve as an early indicator of systemic vulnerability. Recognizing sarcopenia as a modifiable and measurable risk factor opens avenues for early risk stratification and targeted preventive strategies.

To our knowledge, this is the first study to demonstrate an association between possible sarcopenia and the onset of delirium specifically in patients with acute stroke. Our findings are consistent with previous reports showing a link between sarcopenia and delirium in older adults admitted to acute geriatric wards [15]. Additionally, calf circumference, a simple surrogate marker of muscle mass, has also been reported to be associated with delirium onset in older hospitalized patients [16]. By focusing on a stroke-specific population (known to be at particularly high risk for both sarcopenia and delirium), this study provides novel, disease-specific evidence and supports the inclusion of sarcopenia screening in the early evaluation of stroke patients.

Limitations

Several limitations must be acknowledged. Although various factors such as polypharmacy, number of comorbidities, Foley catheter use, pain, nutritional status, and physical restraints may influence the onset of delirium, it was not statistically appropriate to include all of these potential confounders in our multivariate analysis due to sample size constraints. To maintain the validity of the logistic regression model, we prioritized a limited number of covariates based on their clinical relevance and established association with delirium. Therefore, we selected key variables from the Japanese national “Delirium High-Risk Care Fee” checklist, which is widely used in clinical settings. In addition, frailty is a well-recognized clinical construct that overlaps significantly with both sarcopenia and delirium. Although frailty was not formally assessed in this study, it may underlie both conditions and contribute to the observed association. Future studies should consider incorporating validated frailty assessment tools and other relevant factors to further clarify these complex interrelationships.

Secondly, this was a retrospective study conducted at a single center, which may limit the generalizability of the findings. Future multicenter, prospective studies are warranted to validate our findings and further clarify the temporal relationships between sarcopenia and delirium onset. Third, the diagnosis of sarcopenia was based solely on grip strength and calf circumference, excluding the physical performance component required by the full AWGS2019 criteria. This was due to the frequent presence of motor deficits in acute stroke patients, which makes physical performance testing difficult during the early phase of hospitalization. While this pragmatic approach is justified in this context, it may reduce diagnostic precision and comparability across studies. Fourth, the timing of delirium onset was not considered in the analysis. In clinical practice, the impact of delirium often depends on its timing relative to treatment milestones. For example, in patients undergoing endovascular therapy, delirium on the day of catheterization may significantly influence outcomes due to bleeding risk. Incorporating time-to-event analyses, such as Kaplan-Meier models, may better capture these temporal effects. Fifth, we did not assess the severity of sarcopenia or delirium. Severity scores may enhance statistical power and offer more granular insights, and should be addressed in future research.

Despite these limitations, this study provides important evidence that potential sarcopenia is a significant and independent risk factor for delirium in older patients with acute stroke. These findings offer a new perspective on early risk stratification and may guide more effective management strategies for delirium prevention in stroke care.

## Conclusions

From a clinical perspective, our findings underscore the importance of assessing sarcopenia at the time of admission in older patients with acute stroke. Early identification of patients at risk may help facilitate the implementation of multidisciplinary interventions, including nutritional support, mobilization protocols, and delirium prevention strategies. Future prospective studies are warranted to determine whether addressing sarcopenia during hospitalization can reduce the incidence or severity of delirium and improve functional recovery in this vulnerable population.
